# Transcriptional profiling avian beta-defensins in chicken oviduct epithelial cells before and after infection with *Salmonella enterica *serovar Enteritidis

**DOI:** 10.1186/1471-2180-9-153

**Published:** 2009-07-30

**Authors:** Katie L Ebers, C Yan Zhang, M Zhenyu Zhang, R Hartford Bailey, Shuping Zhang

**Affiliations:** 1 Department of Pathobiology and Population Medicine, College of Veterinary Medicine, Mississippi State University, PO Box 97813, Pearl, MS 39288, USA

## Abstract

**Background:**

*Salmonella enterica *serovar Enteritidis (SE) colonizes the ovary and oviduct of chickens without causing overt clinical signs which can lead to SE-contamination of the content and membrane of shell-eggs as well as hatchery eggs. The organism utilizes the *Salmonella *Pathogenicity Island-2 encoded type III secretion system (T3SS-2) to promote persistence in the oviduct of laying hens. In this study, reverse transcriptase-polymerase chain reaction (RT-PCR) was carried out to determine the expression profiles of 14 known avian beta defensins (AvBDs) in primary chicken oviduct epithelial cells (COEC) before and after infections with a wild type SE strain and T3SS mutant SE strains carrying an inactivated *sipA *or *pipB *gene.

**Results:**

Based on the expression levels in uninfected COEC, AvBDs can be loosely grouped into three categories with AvBD4-5 and AvBD9-12 being constitutively expressed at high levels; AvBD1, AvBD3, and AvBD13-14 at moderate levels; and AvBD2 and AvBD6-8 at minimal levels. Infection with the wild type SE strain temporarily repressed certain highly expressed AvBDs and induced the expression of minimally expressed AvBDs. The *pipB *mutant, compared to the wild type strain, had reduced suppressive effect on the expression of highly expressed AvBDs. Moreover, the *pipB *mutant elicited significantly higher levels of the minimally expressed AvBDs than the wild type SE or the *sipA *mutant did.

**Conclusion:**

Chicken oviduct epithelial cells express most of the known AvBD genes in response to SE infection. PipB, a T3SS-2 effector protein, plays a role in dampening the β-defensin arm of innate immunity during SE invasion of chicken oviduct epithelium.

## Background

*Salmonella enterica *serovar Enteritidis (SE) is one of the leading etiologic agents of non-typhoid fever [1]. The disease usually manifests as a self-limiting enteritis, although systemic spread of the infections accompanied by mortalities occurs in young and immunocompromised human patients [2]. Epidemiological studies suggest that poultry flocks may serve as a major reservoir for SE organisms implicated in human clinical cases [3]. *Salmonella enterica *silently colonizes the intestinal and reproductive tracts of chickens, which can provide a mechanism for SE-contamination of chicken meat, shell-eggs, and hatchery eggs if proper processing and handling are not observed [4]. Recent investigations have shown that SE utilizes its type three secretion systems (T3SS) encoded by *Salmonella *pathogenicity island-1 and -2 (SPI-1 and SPI-2), respectively, to promote intestinal and reproductive tract colonization [5-7]. The T3SS of *Salmonellae *functions as a needle-like apparatus that injects an array of effector proteins into host cells. The T3SS-1 effectors act in concert to modulate host cell cytoskeleton rearrangement, thereby facilitating bacterial entry into host epithelial cells [8]. The T3SS-2 effectors promote bacterial survival or replication within host phagocytes [9]. The T3SS effectors also shape the type of pathological changes associated with *Salmonella *infection via modulating host cytokine and chemokine expressions [10].

It has been commonly accepted that the outcomes of microbial infections, including salmonellosis, are largely determined by the type and magnitude of host systemic and local immune responses. At the mucosal surface, antimicrobial peptides, known as defensins, play a key role in preventing *Salmonella *colonization [11]. Defensins are cationic cystein-rich peptides that kill microbial pathogens via multiple mechanisms, such as pore formation and membrane disruption [12-14]. Based on the arrangement of cystein residues, these peptides are further grouped into three subfamilies, namely α-, β-, and θ-defensins [11]. It has been acknowledged that chickens produce only β-defensins, previously known as gallinacins, with 14 avian β-defensin (AvBD) genes being discovered [15-18] The expression of AvBD genes may be influenced by many physiological factors, such as age and breed of the host, as well as the type of tissue or organ tested [19-22]. A recent study suggests that the reproductive tract of laying hens expresses a number of AvBDs and the expression of several AvBDs in vagina epithelium is induced by LPS treatment [23]. Although exposure to LPS mimics certain aspects of bacterial infection in terms of triggering host immune responses, the later is much more complicated and frequently involves the interaction between bacterial virulence factors and specific host cellular pathways. For example, the T3SS of *Bordetella brochiseptica *inhibits NF-KB activation in bovine airway epithelial cells, resulting in the down-regulation of a β-defensin gene, namely TAP [24].

To understand the immunological mechanisms underlying the silent colonization of chicken reproductive tract tissue by SE, we determined the expression profiles of AvBD1 to AvBD14 in primary oviduct epithelial cells prepared from the isthmus of laying hens. We also determined the changes in AvBD expression levels following infections with wild type or T3SS mutant SE strains [25].

## Results

### Intracellular bacterial load and SE-induced COEC apoptosis

**Figure 1:**
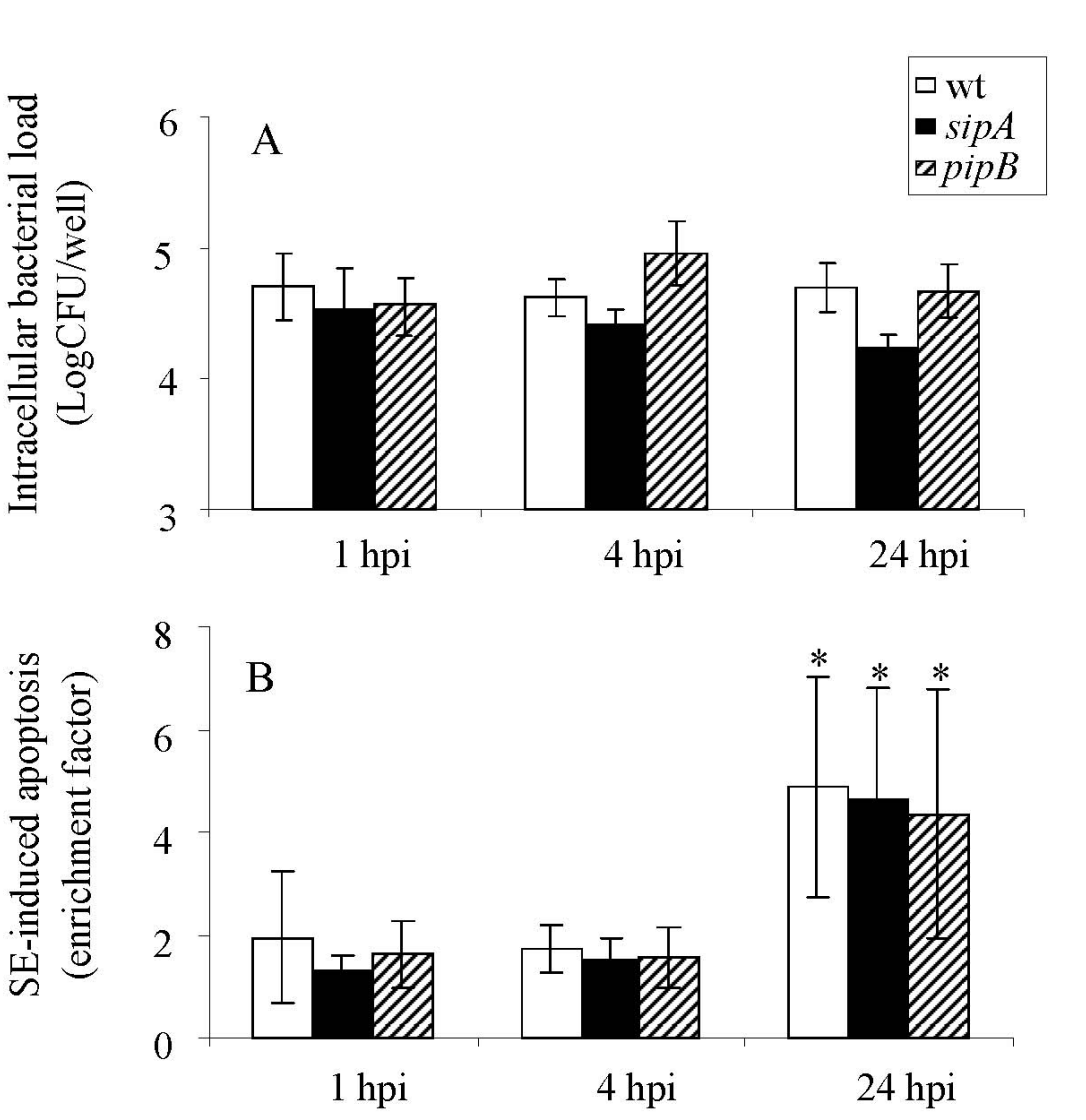
**SE invasion of COEC and induction of COEC apoptosis**. COEC in 48-well culture plates were infected with ZM100 (wt) or ZM106 (*pipB*) at MOI of 20–30:1. 1A. Number of intracellular bacteria presented as log CFU/well. 1B. Apoptosis of COEC expressed as enrichment factor of mono- and oligonucleosomes in the cytoplasm of COEC. Results shown are geometric means of three independent experiments ± standard deviation. Open bar, ZM100 (wt); solid bar, ZM103 (*sipA*); hatched bar, ZM106 (*pipB*). * indicates a significant increase in COEC apoptosis between 1 hpi and 24 hpi (p < 0.05).

Our previous data revealed that SE strains carrying a mutation in *sipA *(ZM103) or *pipB *(ZM106) were less invasive than their wild type parent strain, ZM100. To achieve similar numbers of intracellular bacteria, COEC cultures were initially infected with mutant strains at a higher multiplicity of infection (MOI) than that for the wild type SE. The data showed that comparable numbers of ZM100 (wt), ZM103 (*sipA*), and ZM106 (*pipB*) entered into COEC cultures at 1 hour post infection (hpi) (Figure 1A). Although spontaneous apoptosis of COEC was minimal within the time frame and the experimental conditions used in this study, SE-infections resulted in significant COEC death between 1 hpi and 24 hpi (Figure 1B). However, there was no difference in the degree of apoptosis between COEC cultures infected with the wild type strain and that with the mutants (Figure 1B).

### AvBD expression in primary COEC cultures

**Figure 2:**
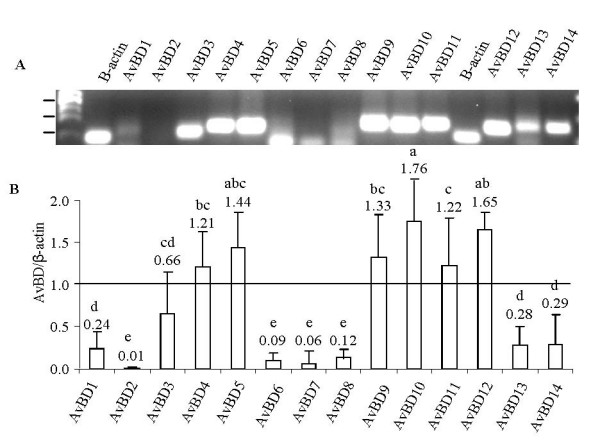
**Expression patterns of 14 known AvBDs in COEC**. Total RNA extracted from uninfected COEC was reverse transcribed into cDNA and amplified using primers specific for β-actin and individual AvBD genes. 2A. RT-PCR products subjected to 1.5% agarose gel electrophoresis. 2B. Amount of AvBD RT-PCR products relative to that of β-actin products. Data presented are geometric means of four independent experiments ± standard deviation. Values with different letters are significantly different (P < 0.05).

To determine the base-level expression of each AvBD gene, RT-PCR assays were performed using total RNA extracted from COEC cultured for 48–72 h. Specific amplification of each AvBD gene was confirmed by sequencing the corresponding PCR products. Electrophoresis of AvBD RT-PCR products revealed three general expression patterns with AvBD4, AvBD5, and AvBD9-12 being constitutively expressed at relatively high levels (ratio of AvBD/β-actin >1); AvBD1, AvBD3, AvBD13-14 at moderate levels (ratio of AvBD/β-actin < 1, but consistently detectable); and AvBD2 and AvBD6-8 at minimal levels (inconsistently detectable) (Figure 2). To determine whether the minimal level expressions of AvBD2, and AvBD6-8 resulted from the use of inadequate PCR primers, alternative primers and PCR conditions were used to amplify these genes which yielded similar results (data not shown). Thus the primers listed in Table 1 were used in subsequent RT-PCR assays.

**Table 1:** Primers used to amplify AvBD and chicken beta-actin genes

Primer	Sequence	Amplicon size
Actin-F Actin-R	5'-tgcgtgacatcaaggagaag-3' 5'-gaccatcagggagttcatagc-3'	111 bp

AvBD-1-F AvBD-1-R	5'-cgaaagagtggcttctgtgc-3' 5'-ggtgatgtcctgcttggg-3'	156 bp
AvBD-2-F AvBD-2-R	5'-aggtttctccagggttgt-3' 5'-tgcattccaaggccattt-3'	146 bp
AvBD-3-F AvBD-3-R	5'-ccactcagtgcagaataagag-3' 5'-aattcagggcatcaacctc-3'	131 bp
AvBD-4-F AvBD-4-R	5'-catctcagtgtcgtttctctgc-3' 5'-cgcgatatccacattgcatg-3'	157 bp
AvBD-5-F AvBD-5-R	5'-ctgccagcaagaaaggaacctg-3' 5'-gtaatcctcgagcaagggaca-3'	155 bp
AvBD-6-F AvBD-6-R	5'-aggatttcacatcccagccgtg-3' 5'-cgacatggcccaggaatgcag-3'	156 bp
AvBD-7-F AvBD-7-R	5'-tggagaagggagacagaaggc a-3' 5'-cgaagcctacaagtatcaat-3'	177 bp
AvBD-8-F AvBD-8-R	5'-acagtgtgagcaggcaggaggga-3' 5'-gaagagctgcttagctggtct-3'	153 bp
AvBD-9-F AvBD-9-R	5'-gcaaaggctattccacagcag-3' 5'-ggagcacggcatgcaacaa-3'	167 bp
AvBD-10-F AvBD-10-R	5'-tggggcacgcagtccacaac-3' 5'-catgccccagcacggcagaa-3'	157 bp
AvBD-11-F AvBD-11-R	5'-actgcatccgttccaaagtctg-3' 5'-gtcccagctgttcttccag-3'	168 bp
AvBD-12-F AvBD-12-R	5'-cccagcaggaccaaagcaatg-3' 5'-agtacttagccaggtattcc-3	157 bp
AvBD-13-F AvBD-13-R	5'-catcgttgtcattctcctcctc-3' 5'-ggtggagaacctgcagcagcg-3'	163 bp
AvBD-14-F AvBD-14-R	5'-atgggcatattcctcctgt-3' 5'-cactttgccagtccattg t-3'	161 bp

### SE-induced changes in AvBD expression in COEC

**Figure 3:**
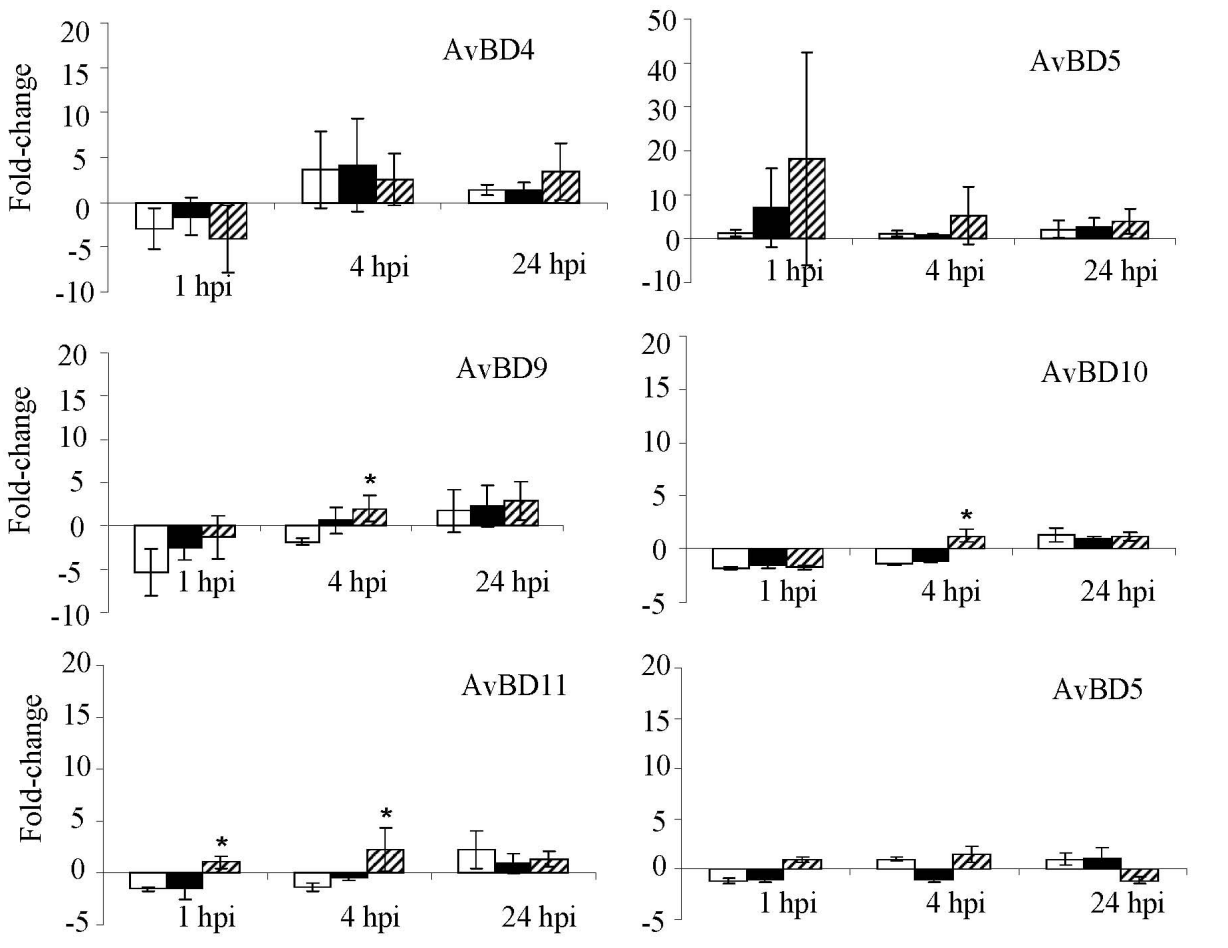
**Transcriptional changes of constitutively and highly expressed AvBDs in COEC following infections with SE**. Data shown (fold change) are geometric means of three independent experiments ± standard deviation. Open bar, ZM100 (wt); solid bar, ZM103 (*sipA*); hatched bar, ZM106 (*pipB*). * indicates that the difference between the transcriptional changes induced by the wild type SE and the mutant is significant (p < 0.05).

**Figure 4:**
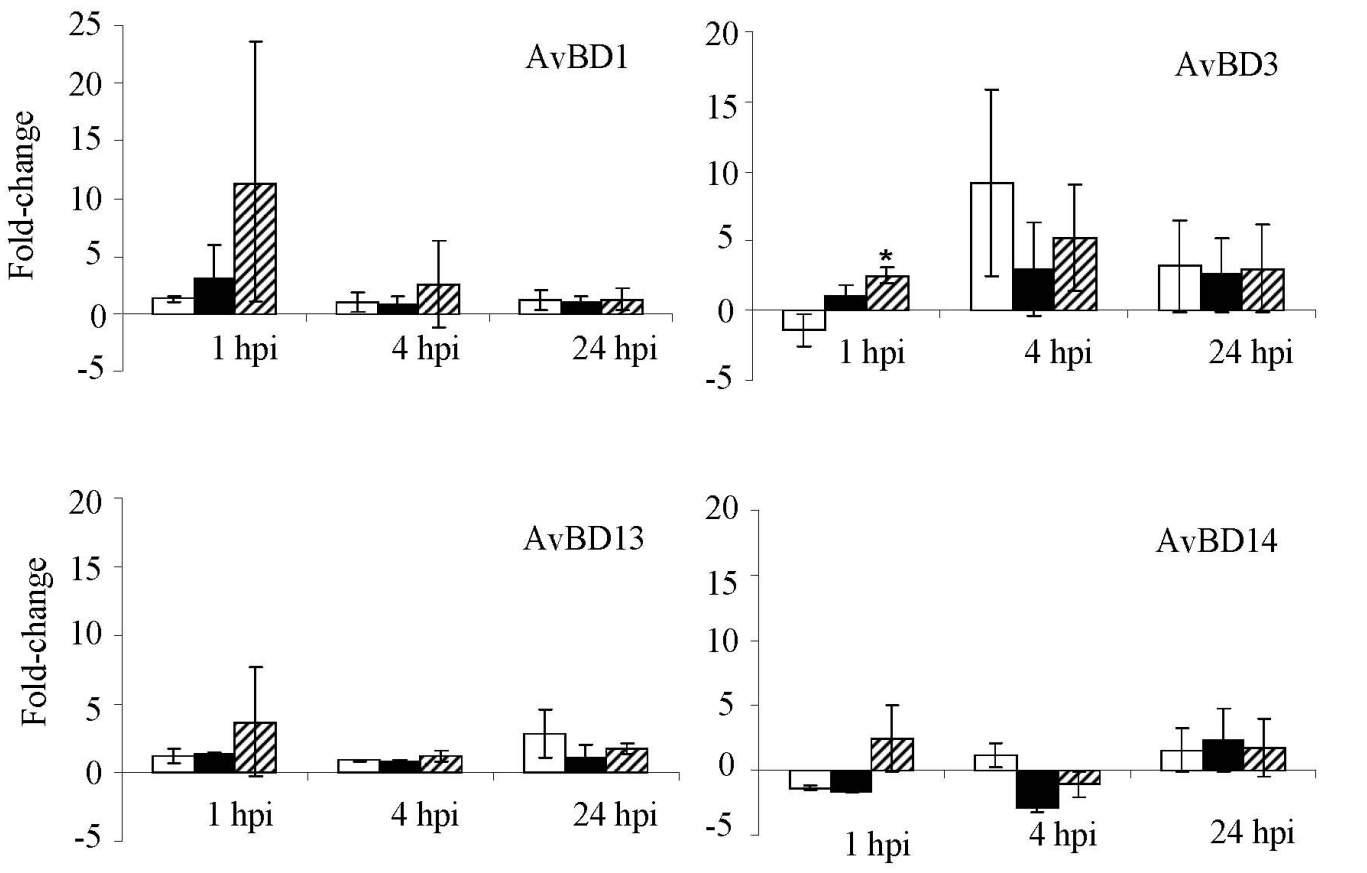
**Transcriptional changes of moderately expressed AvBDs in COEC following infections with SE**. Data shown (fold change) are geometric means of three independent experiments ± standard deviation. Open bar, ZM100 (wt); solid bar, ZM103 (*sipA*); hatched bar, ZM106 (*pipB*). * indicates that the difference between the transcriptional changes induced by the wild type SE and the mutant is significant (p < 0.05).

**Figure 5:**
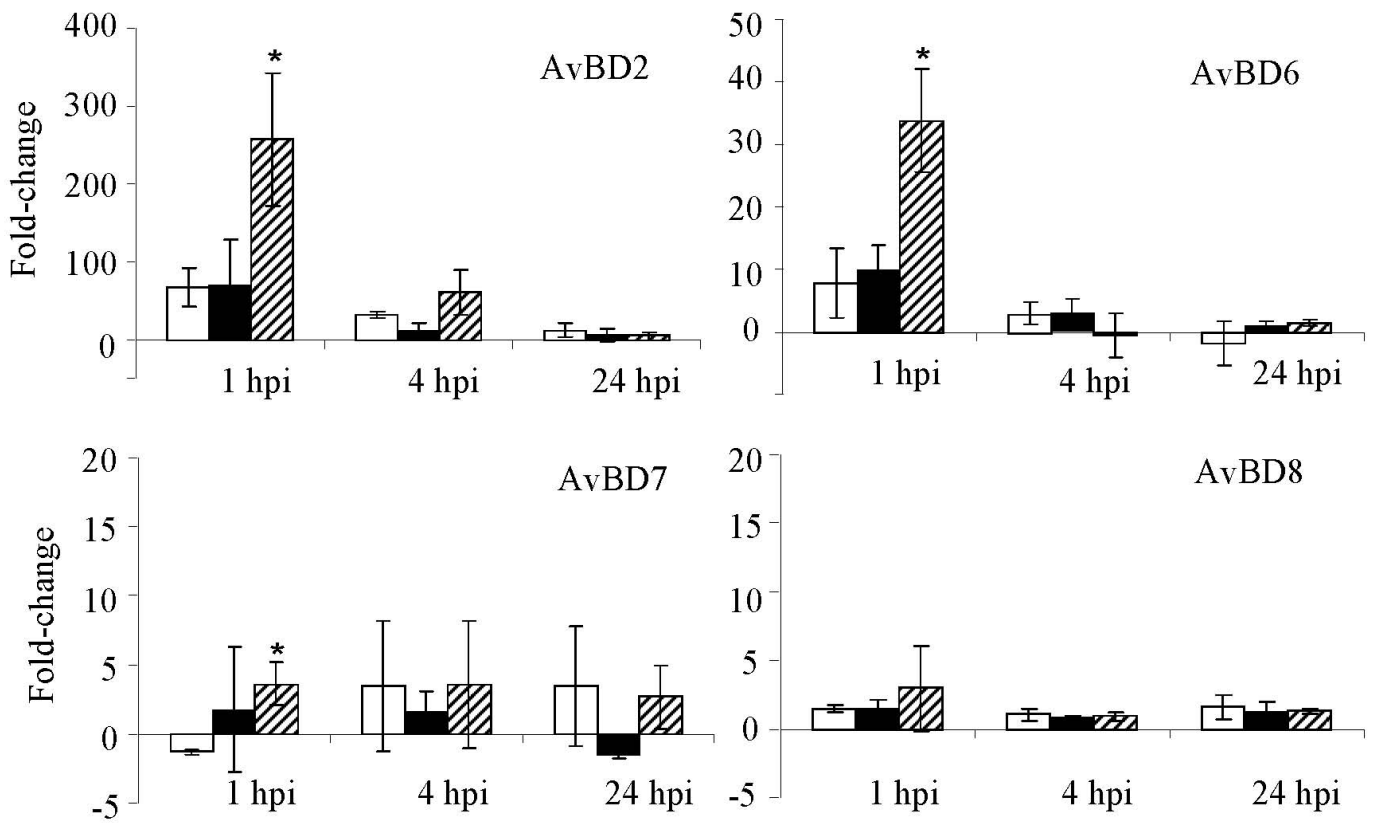
**Transcriptional changes of minimally expressed AvBDs in COEC following infections with SE**. Data shown (fold change) are geometric means of three independent experiments ± standard deviation. Open bar, ZM100 (wt); solid bar, ZM103 (*sipA*); hatched bar, ZM106 (*pipB*). * indicates that the difference between the amounts of AvBD transcripts in ZM100-infected COEC and ZM106-infected COEC is significant (p < 0.05).

Quantitative real-time RT-PCR analysis of AvBD expression profiles in SE-infected COEC cultures indicated that SE-induced transcriptional changes mostly occurred at 1 hpi and to a certain extent, at 4 hpi (Figure 3, 4 and 5). Differential induction of certain AvBDs by the wild type SE and the *pipB *mutant was also observed at these times. Among the constitutively and highly expressed AvBD genes, infection of COEC with ZM100 (wt) or ZM103 (*sipA*) resulted in a temporary repression of AvBD4, and AvBD9-11 (≤ 1.5-fold), but not AvBD5 and AvBD12 (Figure 3). Infection of COEC with ZM106 (*pipB*) had reduced or no suppressive effect on the transcription of AvBD9-11, compared to infections with strains ZM100 and ZM103 (Figure 3). With the moderately expressed genes, infection of COEC with ZM100 (wt) or ZM103 (*sipA*) had minimal effect (< 1.5-fold) on the expression of AvBD1 and AvBD13-14, whereas ZM106 (*pipB*) temporarily induced the expressions of these genes at 1 hpi (Figure 4). The expression of another moderately expressed gene, namely AvBD3, was initially suppressed by ZM100, but not ZM106, and then induced by all three SE strains at 4 hpi and 24 hpi (Figure 4). With the minimally expressed genes, AvBD2 and AvBD6 were induced by all SE strains examined. However, the expression levels of AvBD2 and AvBD6 in COEC infected with ZM106 were significantly higher than that in COEC infected with ZM100 or ZM103 (Figure 5). The expression of AvBD7 and AvBD8 in COEC was minimally affected by exposures to ZM100 and ZM103. Compared to the wild type strain and the *sipA *mutant, ZM106 also induced elevated expression of AvBD7 (Figure 5).

### Verification of *pipB*-dependent suppression of AvBD in SE-infected COEC

**Figure 6:**
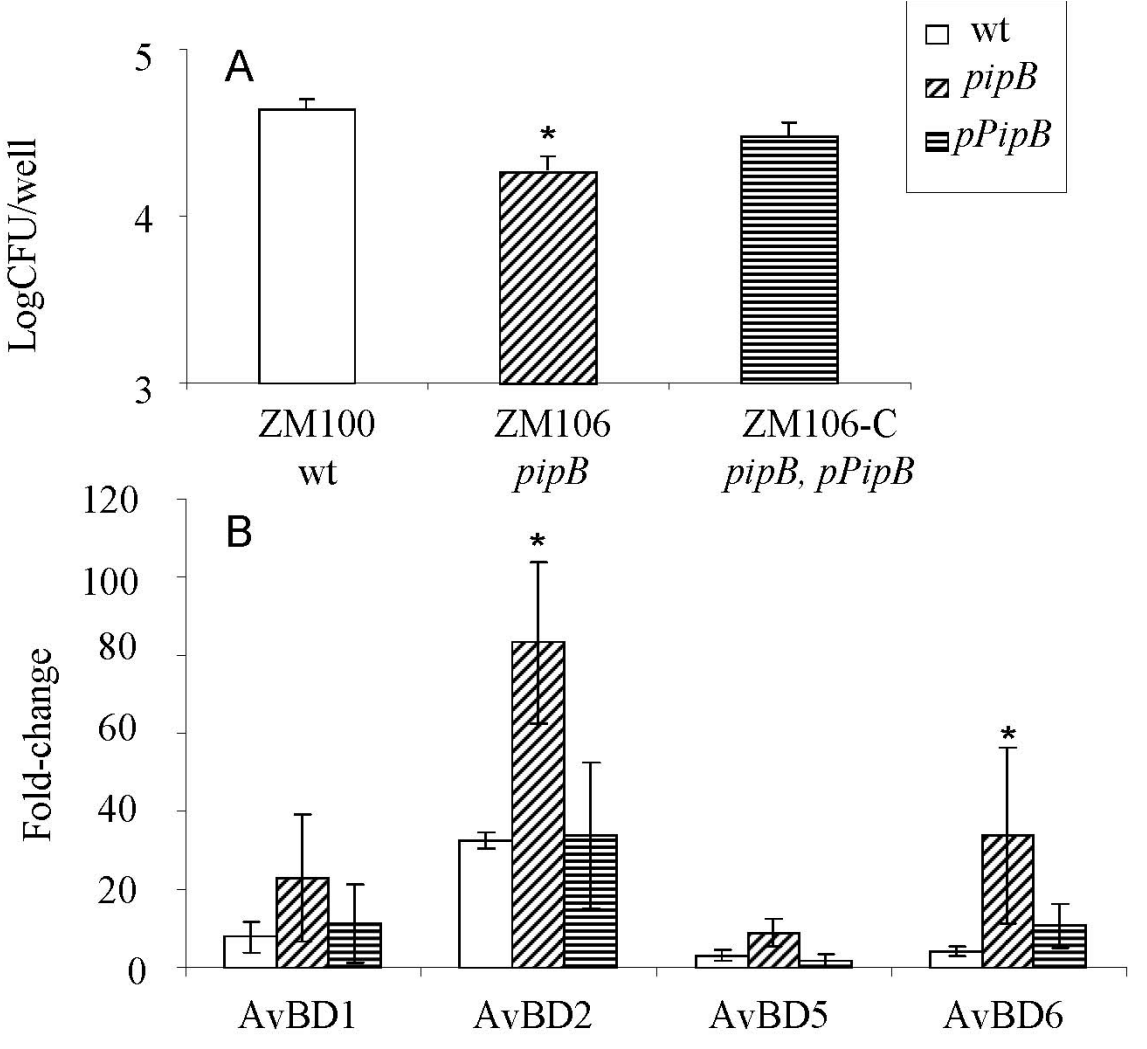
**PipB-mediated entry of SE into COEC and suppression of AvBDs in SE-infected COEC**. COEC in 48-well culture plates were infected with ZM100 (wt), ZM106 (*pipB*), or ZM106-C (*pipB, pPipB*) at MOI of 20:1 (bacteria:cell). Data shown are geometric means of three independent experiments ± standard deviation. 6A. Number of intracellular bacteria (log CFU/well) at 1 hpi. * indicates that the difference in the number of intracellular bacteria between ZM100 (wt) and ZM106 (*pipB*) is significant (p < 0.05). 6B. SE-induced changes in the mRNA expression of AvBDs in COEC at 1 hpi. * indicates that the difference between the amounts of AvBD transcripts in ZM100-infected COEC and ZM106-infected COEC is significant (p < 0.05).

To rule out the possibility that increased AvBD expression in COEC infected with ZM106 was caused by the insertion of a chloramphenicol resistance cassette in the chromosome of this strain, COEC cultures were infected with ZM100, ZM106, or ZM106-C at the same MOI (20:1). The number of bacteria that entered into COEC and the expression of selected AvBD genes were determined at 1 hpi. The results showed that ZM106 (*pipB*) was less invasive than ZM100 (wt) and introduction of pPipB, a plasmid expressing the *pipB *gene, to ZM106 (*pipB*) complemented the invasion defect of this strain (Figure 6A). Although the number of ZM106 that entered into COEC was less than that of the wild type SE, ZM106 still induced the expression of AvBD2 and AvBD6 at levels higher than that induced by ZM100 (Figure 6B). Introduction of the cloned *pipB *gene into ZM106 weakened the strain's capacity to induce AvBD mRNA expression (Figure 6B). Thus, differential induction of AvBDs by ZM100 and ZM106 was indeed associated with their genetic backgrounds, with or without a functional *pipB*.

## Discussion

As a key component of innate immune response, defensins are synthesized in many tissues, especially those constantly exposed to microbial pathogens [26-30]. For example, a number of AvBD genes are expressed in the vagina of laying hens and the amount of AvBD mRNA increases following LPS treatment [31]. Although the vagina is anatomically prone to exposure to intestinal or environmental pathogens, the isthmus is likely a critical site in terms of persistent reproductive tract colonization and egg membrane contamination by SE [32,33]. In an attempt to understand the innate immune responses against SE colonization of chicken oviduct epithelium, we determined the AvBD expression profile in primary oviduct epithelial cells. Although the preparation of primary chicken oviduct epithelial cells is empirical, the COEC cultures used in this study consisted of a high percentage of epithelial cells and spontaneous apoptosis of COEC was minimal under the experimental conditions used. Because chicken epithelial cell lines are not available at the present time, the primary COEC model is a useful tool in studying the early interactions between SE and chicken oviduct epithelium. Our results showed that AvBD1, AvBD3-5, and AvBD9-14 were constitutively expressed at moderate or high levels in the isthmal epithelial cells of laying hens. Our data differed from previous findings with regard to the expression of several AvBDs. First, one report showed that AvBD1-7 was mainly expressed in bone marrow whereas AvBD8-13 were restricted in the urogenital tract of young hens [18]. Second, another study indicated that most AvBDs, except AvBD6 and AvBD13, were expressed in all segments of oviduct of White Leghorn laying hens [23]. Tissue-specific expression of AvBD14, a newly discovered avian β-defensin, has not been previously reported. Given that the adequacy of PCR primers and conditions as well as the specificity of RT-PCR products being confirmed in the present study, the discrepancies between our results and others' may reflect the differences between the experimental conditions, such as the breeds of hens (Ross versus White Leghorn) and the sources of RNA (cultured oviduct epithelial cells versus oviduct tissue). It is plausible that the different AvBD expression profiles presented by various investigators suggest a complex regulatory mechanism(s) governing the expression of AvBD genes in different types of hosts, tissues, or even cells.

AvBDs play significant roles in host resistance to *Salmonella *colonization as indicated by the correlation between a high level expression of AvBD and a low level of *Salmonella *load in the caecum [19,21]. Either LPS treatment or *Salmonella *infection can induce the expression of certain AvBD genes in chicken reproductive tissues [22,31,34]. In this study, SE temporarily modulated the expression of certain AvBDs in the early stages of infection. Increased apoptosis of COEC may be partially responsible for the decline in SE-induced expression of certain AvBDs, such as AvBD2 and AvBD6, but it does not explain the diminished suppression of AvBD4 and AvBD9-11 by SE in the late stage of infection. We therefore hypothesize that SE-modulation of AvBD transcription involves tightly controlled signaling events that take place during the initial interaction between COEC and SE. In mammalian hosts, recognition of pathogen-associated molecular pattern (PAMP) by toll-like receptors (TLR) activates nuclear factor kappa B (NF-κB) and mitogen-activated protein kinase (MAPK), leading to the up-regulation of beta defensin-2 [35]. Thus, it is likely that LPS, flagellin, and/or secreted virulence factors of SE function as PAMP to trigger the expression of AvBDs in COEC. We also observed that inactivation of *pipB*, a gene encoding a T3SS translocated protein, increases the ability of SE to stimulate AvBD expression in COEC. The differential induction of AvBDs by ZM100 and ZM106 was only observed when AvBDs were maximally induced (or repressed) by the wild type strain at 1 hpi and/or 4 hpi. The three different facts lead us to believe this phenomenon was apparently not a result of altered COEC viability or the presence of a chloramphenicol resistance cassette (pEP185.2) in ZM106 were 1) both the wild type and mutant SE strains induced similar degrees of COEC apoptosis; 2) ZM103 (*sipA*) carrying the same chloramphenicol resistance cassette displayed a wild type phenotype in terms of modulating AvBD expression; and 3) introduction of the cloned *pipB *gene into ZM106 reduced the strain's ability to induce AvBD expression. One possible explanation for the elevated induction of AvBDs by ZM106 (*pipB*) may be that PipB interferes with one or more steps of the signaling pathway leading to the activation of AvBD genes, such as PAMP-TLR-NFkB/MAPK-AvBD promoter. At the present time, the role of *pipB *in the pathogenesis of salmonellosis is not well understood. Limited data indicates that *pipB *is a chicken host-specific colonization factor of *Salmonella enterica *serovar Typhimurium [36]. PipB is targeted to detergent-resistant microdomains of intracellular membranes, which lead to the speculation of a possible interaction between PipB and host cell signaling molecules [37]. Our recent investigation found that *pipB *is required by SE to invade COEC and survive within peripheral blood lymphocyte derived monocytes [25]. Although the mechanism of action remains to be elucidated, data from the present study reveals a *pipB*-mediated inhibition of AvBD expression in SE-infected COEC, another strategy used by SE to weaken host innate immunity in the oviduct epithelium of laying hens. However, the biological significance of PipB-mediated alterations in AvBD expression should be further evaluated using in vivo infection models.

## Conclusion

Data from study indicates that the oviduct epithelial cells of laying hens constitutively express most AvBDs, except AvBD2 and AvBD6-8, at moderate to high levels in comparison to the expression of β-actin. SE briefly suppresses the transcription of several constitutively and highly expressed AvBDs and stimulates the expression of minimally expressed AvBDs in COEC. PipB, a T3SS-2 effector protein, plays a role in repressing AvBD genes during SE invasion of COEC.

## Methods

### Bacterial strains and growth conditions

A spontaneous nalidixic acid-resistant strain of SE, ZM 100 (wt), and its isogenic mutants, ZM103 (*sipA*) and ZM106 (*pipB*) were grown aerobically in tryptic soy agar or broth supplemented with nalidixic acid at a concentration of 50 μg/ml at 37°C [25]. To prepare the inoculum, 50 μl of an overnight culture of each bacterial strain was diluted into 5 ml of fresh TSB and incubated aerobically for 4 hours (h) at 37°C. Cultures of SE at the logarithmic phase of growth were harvested by centrifugation at 1,500 × *g *for 15 min and re-suspended in fresh HBSS without antibiotics. The number of bacteria in each culture was determined by measuring the density at OD600 and confirmed by subsequent CFU enumerations.

### Cell culture and culture condition

Primary chicken oviduct epithelia cells (COEC) were prepared similarly to those described previously [32]. The oviduct tissues of 25–28 week old broiler breeder hens (Ross) were obtained from a commercial processing company. The isthmal epithelium of the oviduct was washed extensively with HBSS containing 200 U/ml penicillin and 200 mg/ml streptomycin and treated with 20 ml of HBSS containing 1 mg/ml collagenase (Sigma) for 30 min at 37°C. Following collagenase treatment, the supernatant was discarded and the tissue fragments were digested three times with 0.25% trypsin and 3 mM EDTA in 20 ml of HBSS for 10 min at 37°C. The cells suspension was supplemented with 10% of heat-inactivated fetal bovine serum (FBS) to stop the activity of trypsin. To remove undigested tissue clumps, the cell suspension was passed through cell strainers (100-micro pores). To separate epithelial cells, which quickly formed cell aggregates, from erythrocytes, platelets, and other immune cells, the cell suspension was centrifuged at 50 × *g *for 5 min. Following centrifugation, supernatant containing fibroblasts, erythrocytes, and immune cells, was discarded and the loose pellet containing epithelial cells and cell sheets was resuspended in 20 ml of HBSS. After three low-speed centrifugations, the cell pellet was resuspended in minimal essential medium (MEM, ATCC) supplemented with 10% FBS, 2% heat-inactivated chicken serum (CS), insulin (0.12 U/ml), and estradiol (50 nM). The COEC cells were incubated in Petri dishes for 2 h at 39°C in 5% CO_2 _to allow fibroblast cells to attach. Following incubation, epithelial cells were collected by gentle pipetting and subsequent centrifugation at 125 × *g *for 10 min. The pelleted epithelial cells were resuspended in fresh MEM medium and seeded into 48-well tissue culture plates at a density of approximately 8 × 10^4 ^cells per well and incubated for 24 h to 48 h at 39°C in 5% CO_2 _until infection took place.

### Immunohistochemistry

COEC cultures were incubated with monoclonal anti-pan cytokeratin mouse Ab (epithelial cell marker) for 2 h at 37°C, washed three times, then incubated with fluorescein isothiocyanate (FITC) anti-mouse IgG for 1 h at 37°C. Staining of cytoskeleton of COEC was viewed with an Olympus IX81 FA scope. Cultures with more than 80% of cytokeratin-positive cells were used in subsequent infections. Thus, the COEC preparations consisted of more than 80% epithelial cells, less than 20% fibroblast, and possibly residual amount of immune cells.

### Infection of cell culture

Infections were conducted using the gentamicin protection method as described previously [25]. Prior to inoculation, cell cultures were washed 3 times with pre-warmed Hanks' Balanced Salt Solution (HBSS) without antibiotics. For each bacterial strain/time point combination, 500 μl of bacterial suspension containing approximately 16 to 24 × 10^5 ^CFU was added into each of the six wells to reach a multiplicity of infection (MOI) of 20:1 to 30:1 (bacteria:cells). The inoculated cell cultures were centrifuged at 800 × *g *for 10 min and then incubated for 1 h at 39°C in 5% CO_2_. To remove extracellular bacteria, the infected cell cultures were washed 3 times with pre-warmed HBSS and incubated in 500 μl of HBSS containing gentamicin at a concentration of 100 μg/ml for an additional hour at 39°C in 5% CO_2_. After incubation, the infected cells were either lysed by incubating with TRIzol for RNA extraction or with 0.2% Triton X-100 for bacterial CFU enumeration which was designated as 1 hpi. The remainders of the COEC cultures were maintained in supplemented MEM containing 50 μg/ml gentamicin for an additional 3 h and 23 h followed by cell lysis. These later time points were designated as 4 hpi and 24 hpi, respectively. Ten-fold dilutions of the original inoculum and cell lysate were plated onto tryptic soy agar (TSA, Difco) plate supplemented with 50 μg/ml of nalidixic acid and incubated overnight at 37°C for bacterial CFU enumerations.

### Cell Death Detection ELISA

SE-induced apoptosis of COEC was evaluated using the Cell Death Detection ELISA plus system (Roche). Briefly, SE-infected and uninfected COEC cultures were treated with the lysis buffer for 30 min at room temperature and centrifuged at 200 × *g *for 10 min. One tenth of the cell lysate was transferred to the streptavidin-coated microplate and incubated with anti-histone and anti-DNA antibodies for 2 h at room temperature. The antibody-nucleosome complexes bound to the microplates were incubated with peroxidase substrate for 15 min at room temperature. The absorbance at 405 nm was then determined. SE-induced apoptosis, expressed as an enrichment factor of mono- and oligonucleosomes in the cytoplasm of COEC, was calculated according to the formula: (absorbance of the infected COEC) – (absorbance of the background)/(absorbance of control COEC) – absorbance of the background). Experiments were repeated 3 times with replicate wells for each treatment group at each time point. Data generated from three independent experiments were presented as mean ± S.D.

### Reverse transcriptase polymerase chain reaction (PT-PCR)

Total RNA was extracted from control and SE-infected COEC cultures at 1 hpi, 4 hpi, and 24 hpi using TRIzol reagent according to the manufacturer's instructions (Life Technologies). Real-time PCR was conducted using MultiScribe reverse transcriptase (Invitrogen) and the DNA labeling dye SYBR Green (Applied Biosystems) as previously described [1]. The primer sequences of chicken β-actin and 14 AvBD genes were obtained from the Entrez Nucleotide database and listed in Table 1. Reverse transcription of total RNA (2 μg) in a mixture containing 100 μl of 5.5 mM MgCl2, 500 μM dNTP, 2.5 μM random hexamers, and 1.25 U of MultiScribe reverse transcriptase per μl was performed at 48°C for 30 min. Real-time PCR was performed using each cDNA product as a template (4 μl/reaction) in duplicates by using gene-specific primers (300 nM) and an ABI Prism 7700 thermocycler (95°C for 10 min followed by 45 amplification cycles of 95°C for 15 s and 58°C for 30 sec and 72°C). The volume of PCR reaction was 25 μl. To determine the base-levels of AvBD transcripts in control COEC, amplified products were subjected to 1.5% agarose gel electrophoresis followed by image capture using an AlphaImager™ 3400. The average intensity of each PCR product with correct size was measured and the ratio between AvBD and β-actin PCR products was calculated. Expression values were caculated using the comparative Ct method as described by Applied Biosystems (User Bulletin No. 2). The threshold cycle (Ct) represents the cycle number at which the amount of amplified target reaches a fixed threshold. For the convenience of calculation, the default upper limit PCR cycle number [45] was assigned to reactions that failed to detect a signal (no amplification). The Ct values of AvBDs were subtracted by the Ct value of β-actin (internal control) of the same sample. The normalized Ct values of AvBD genes amplified from SE-infected COEC relative to that of the control COEC at each time point was calculated as the fold-change using the formula 2^-ΔΔCt ± SD ^where SD is the standard deviation.

### Statistical analysis

Differences in the number of intracellular bacteria and the levels of AvBD expression induced by wild type and mutant SE strains were determined by performing a two-tail Student *t *test (P < 0.05).

## Abbreviations

AvBD: avian beta defensin; COEC: chicken oviduct epithelial cell; h: hour; hpi: hour post infection; HBSS: hanks balanced salt solution; RT-PCR: reverse transcriptase-polymerase chain reaction; SE: *Salmonella enterica *serovar Enteritidis; PAMP: pathogen-associated molecular pattern; T3SS: type three secretion system; TLR: toll like receptor;

## Authors' contributions

KLE performed cell culture, RNA extraction, and RT-PCR. CYZ performed RT-PCR and data analysis. MZ, HB, and SZ drafted the manuscript. All authors read and approved the final manuscript.
